# Purine Intake and All-Cause Mortality in Ovarian Cancer: Results from a Prospective Cohort Study

**DOI:** 10.3390/nu15040931

**Published:** 2023-02-13

**Authors:** Zongda Du, Tingting Gong, Yifan Wei, Gang Zheng, Junqi Zhao, Bingjie Zou, Xue Qin, Shi Yan, Fanghua Liu, Qian Xiao, Qijun Wu, Song Gao, Yuhong Zhao

**Affiliations:** 1 Department of Clinical Epidemiology, Shengjing Hospital of China Medical University, No. 36 Sanhao Street, Shenyang 110001, China; 2 Clinical Research Center, Shengjing Hospital of China Medical University, No. 36 Sanhao Street, Shenyang 110001, China; 3 Liaoning Key Laboratory of Precision Medical Research on Major Chronic Disease, Shengjing Hospital of China Medical University, No. 36 Sanhao Street, Shenyang 110001, China; 4 Department of Obstetrics and Gynecology, Shengjing Hospital of China Medical University, No. 36 Sanhao Street, Shenyang 110001, China; 5 Key Laboratory of Reproductive and Genetic Medicine (China Medical University), National Health Commission, Shenyang 110001, China

**Keywords:** cohort study, diet, mortality, ovarian cancer, purine

## Abstract

Background: Current biological evidence suggests that purine involvement in purine metabolism may contribute to the development and progression of ovarian cancer (OC), but the epidemiological association is currently unknown. Methods: A total of 703 newly diagnosed patients with OC aged 18–79 years were included in this prospective cohort study. Utilizing a verified food-frequency questionnaire, the participants’ dietary consumption was gathered. Using medical records and ongoing follow-up, the deaths up until 31 March 2021 were determined. To assess the hazard ratios (HRs) and 95% confidence intervals (CIs) of purine intake with OC mortality, Cox proportional-hazard models were utilized. Results: During the median follow-up of 31 months (interquartile: 20–47 months), 130 deaths occurred. We observed an improved survival for the highest tercile of total purine intake compared with the lowest tercile (HR = 0.39, 95% CI = 0.19–0.80; *p* trend < 0.05), and this protective association was mainly attributed to xanthine intake (HR = 0.52, 95% CI = 0.29–0.94, *p* trend < 0.05). Additionally, we observed a curving relationship in which OC mortality decreased with total purine intake, and the magnitude of the decrease was negatively correlated with intake (*p* non-linear < 0.05). Significant inverse associations were also observed in subgroup analyses and sensitivity analyses according to demographic and clinical characteristics. Moreover, we observed that xanthine intake and hypoxanthine intake had a multiplicative interaction with ER and PR expression (*p* < 0.05), respectively. Conclusion: A high total purine and xanthine intake was linked to a lower risk of OC mortality. Further clarification of these findings is warranted.

## 1. Introduction

Gynecological cancer is a worldwide public health concern that affects about 10% of women [1]. Ovarian cancer (OC) is the most lethal gynecological cancer [1]. In 2020, OC was diagnosed in 313,959 women worldwide and caused 207,252 deaths [1]. Because this disease is usually asymptomatic or has nonspecific symptoms, more than 75% of patients are diagnosed at an advanced stage [2]. Moreover, the occurrence of chemoresistance also weakens the therapeutic effects of chemotherapeutic treatments [3]. Thus, the 5-year survival rate of OC is below 45% [4]. Although previous research identified a number of prognostic factors for OC, including age at diagnosis [5], tumor stage [6], genotyping [7], and histological type [8], these factors are difficult to change. In recent years, a number of studies focused on finding natural and synthetic compounds that can reduce/prevent chemotherapy resistance in ovarian cancer [9] and ultimately reduce ovarian-cancer mortality. To this end, dietary changes are of great significance to improve the prognosis of OC [10]. Our previous research suggested that dietary factors may play a significant role in the prognosis of OC [11,12,13].

Purine intake is a vital part of the daily diet and plays an essential role in the occurrence and development of cancers through several biological processes [14]. For example, purines are metabolized in the body to produce serum uric acid (SUA), a powerful antioxidant, which might prevent cellular oxidative stress and the occurrence and development of cancers by modifying oxygen radicals’ toxic and cancerous effects [15,16]. Furthermore, previous research suggested that SUA due to purine intake was associated with increased cancer mortality [17]. High SUA due to purine intake seems to support the development of cancer metastasis [18,19].

Previous studies addressed the reasonable biological mechanism that purine involvement in purine metabolism may contribute to the development and progression of OC [20]. However, as far as we are aware, the relationship between dietary purine intake and OC mortality has not been examined in any epidemiological research. Therefore, we conducted this prospective cohort study on 703 OC patients to better elucidate this topic.

## 2. Materials and Methods

### 2.1. Study Population

**Figure 1:**
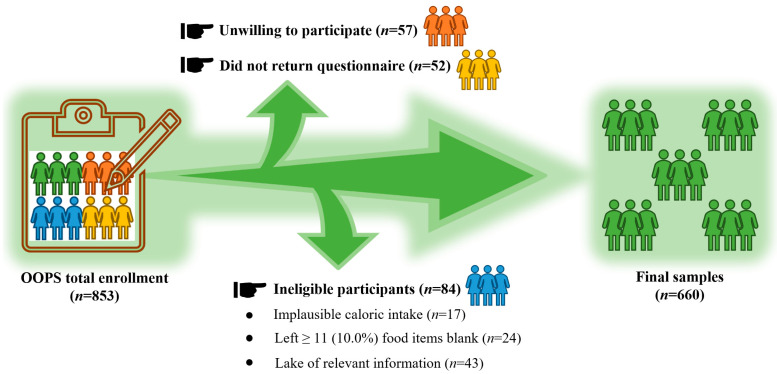
Flowchart outlining inclusion and exclusion in the ovarian-cancer follow-up study (OOPS).

The ovarian-cancer follow-up study (OOPS), a study of prospective longitudinal cohorts [21,22], was carried out in 2015 to gather information on demographics, clinical conditions, lifestyle choices, and dietary patterns and to analyze how these factors relate to the prognosis of OC. The Institutional Review Board of the Ethics Committee of Shengjing Hospital of China Medical University gave its approval to the OOPS (2015PS38K). From January 2015 through December 2020, new cases of OC in women between the ages of 18 and 79 were found. A total of 853 eligible women were found, and 796 (93%) of them provided informed consent to take part in the study, while 744 (87%) submitted the full questionnaire. The following individuals were excluded from our study population: those with unusual caloric intake [23] (<500 or >3500 kcal per day; *n* = 17), and those who omitted 11 (10%) or more dietary items (*n* = 24). After the application of exclusion criteria, 703 women with a diagnosis of OC were included (Figure 1).

### 2.2. Data Collection

At baseline, the sociodemographic information and lifestyles of patients with OC were collected using a standard self-administered questionnaire. In addition, trained staff members measured the participants’ heights and weights in accordance with a prescribed protocol, and weight (kg)/height (m^2^) was used to compute body-mass index (BMI). The clinical information was taken from the electronic medical records of the Shengjing hospital information system. The detailed parameters and grading are provided in the eMethods in the supplementary materials.

#### 2.2.1. Dietary-Exposure Assessment

A validated 111-item food-frequency questionnaire (FFQ) was used to collect dietary data, which was validated previously [24]. The reproducibility coefficients (intraclass correlation coefficients and Spearman correlation coefficients) for the majority of food groups were over 0.5, whereas correlation values (Spearman correlation coefficients) between the FFQ and weighted diet records for the majority of food groups ranged from 0.3 to 0.7 [13,22]. Seven predetermined types of frequency that ranged from “rarely” to “2 or more times per day” were used to determine how frequently, on average, participants consumed specific meals throughout the 12 months preceding OC diagnosis. The primary exposure variable we analyzed was total purine (the sum of xanthine, hypoxanthine, adenine, and guanine intake), xanthine, hypoxanthine, adenine, and guanine. The frequency of consumption of each food was multiplied by the number of nutrients in the recommended servings, and the total amount of nutrients in all the foods consumed by each participant was added to determine the daily nutrient intake. Based on information from the Chinese Food Composition Tables, the nutritional levels were calculated [25].

#### 2.2.2. Immunohistochemistry (IHC)

For IHC analysis, we collected tumor and adjacent tissue from OC patients. The specimens were fixed with formalin and embedded in paraffin with a thickness of 3–4 μm. Before being hydrated in an ethanol gradient, the samples were deparaffinized with xylene. Before heat-induced antigen-epitope retrieval in citrate buffer, the sections were quenched in fresh 3% hydrogen peroxide to limit endogenous-tissue-peroxidase activity (pH 6.0). Next, these sections were blocked by normal serum solution and then incubated with a primary antibody against Wilms’ tumour-1 (WT-1, 1:500, Abcam, ab89901), estrogen receptor (ER, 1:500, Abcam, ab32063), progesterone receptor (PR, 1:500, Abcam, ab32085), and vimentin (1:500, Abcam, ab92547) at 4 °C overnight. After washing with PBS, the sections were incubated with the corresponding secondary antibodies for 30 min at 37 °C. Finally, sections were colored and counterstained with diaminobenzidine and hematoxylin. The staining was graded based on the percentage of positively stained cells and the intensity of the staining. All indicators (WT-1, ER, PR, and vimentin) were divided into positive and negative. Two experienced pathologists independently confirmed IHC expression.

### 2.3. Statistical Analysis

Baseline characteristics of total purine intake were descriptive analyses. Mean and standard deviation were used to describe the variables conforming to the normal distribution, while the one-way ANOVA and Kruskal–Wallis test were used to compare variables that did not conform to the normal distribution; the median and interquartile range were employed to describe variables that did not conform to the normal distribution. The chi-squared test was used to compare categorical variables, which were displayed as frequencies with percentages. By comparing the theoretical sample size (*n* = 443) calculated by the sample-size calculation formula for prospective cohort studies with the actual sample size (*n* = 703) in this study, we confirmed that the actual sample size of our study met the requirements and the research results were reliable. A survival curve was plotted using the Kaplan–Meier technique and we evaluated the association between prediagnostic total purine intake and mortality with hazard ratios and 95% confidence intervals calculated by the Cox proportional-hazard regression model. Detailed methods are provided in the attached eMethods.

Additionally, stratified exploratory analyses were performed by using demographic information and clinical information about the participants. The detailed items and grading are provided in the eMethods in the supplementary materials. Potential interactions between the purine intake and all the stratifying variables above were assessed by adding cross-product terms in multivariable regression models, respectively. Furthermore, we calculated both multiplicative and additive interactions to determine whether the effects of total purine and all kinds of purine on mortality changed. Effect modification was investigated for multiplicative interaction by including interaction variables in multivariate-adjusted models. We computed the relative excess risk as a result of the additive interaction (RERI) [26].

In addition, we conducted several sensitivity analyses. First, we excluded participants with follow-up time < 1 year to reduce potential reverse causation. Second, we used the residual method and energy-density method to adjust the intake energy, to eliminate the deviation caused by different energy-adjustment methods to ensure the credibility of the conclusion [27]. Third, participants with dietary changes were removed to eliminate the effect of dietary-pattern changes on results. Lastly, to test for nonlinear relationships between purine consumption and OC survival, a restricted cubic spline model with three knots—the fifth, fifty-fifth, and ninety-fifth—was used [28]. For all tests, a two-sided *p* value 0.05 was regarded as significant. We used SAS version 9.4 (SAS Institute, Cary, NC, USA) to perform all statistical analyses.

## 3. Results

During the median follow-up of 31 months (interquartile: 20–47 months), 130 deaths occurred. Table 1 summarizes the basic characteristics of the patients with OC. The patients with higher total purine intake tended to consume more fatty acids and cholesterol (all *p* < 0.05). In addition, to elucidate the effect of clinical factors on ovarian-cancer mortality, we adjusted only for clinical factors and found that the patients with non-serous histological subtypes, larger residual lesions, and advanced FIGO stage had significantly higher mortality (Supplementary Table S1).

After adjusting for potential confounding factors (Table 2), we found that the mortality of the high-total-purine-intake group was lower than that of the low-total-purine intake group (HR = 0.39, 95% CI = 0.19–0.80, *p* trend < 0.05; Supplementary Figure S1). Additionally, in the analysis of various types of purine, we found that higher xanthine intake was associated with lower mortality (HR = 0.52, 95% CI = 0.29–0.94, *p* trend < 0.05). No statistically significant relationships were observed between other purines and OC mortality.

**Figure 2:**
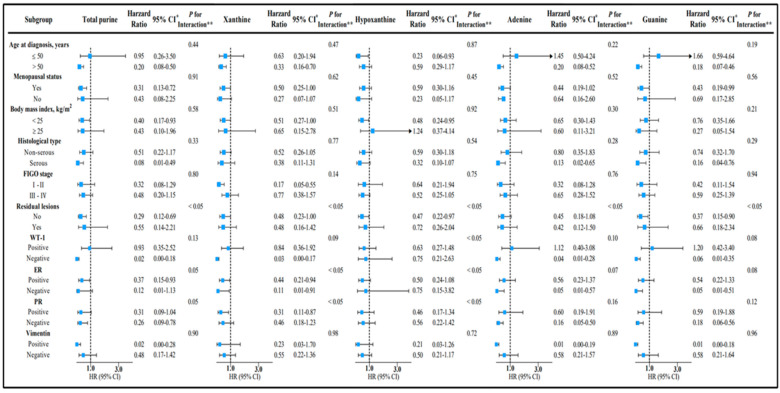
Subgroup analyses of demographic and clinical characteristics for adjusted hazard ratio (HR) and 95% confidence interval (CI) * for the association of dietary purine intake with mortality in ovarian-cancer patients. CI, confidence interval; ER, estrogen receptor; FIGO, The International Federation of Gynecology and Obstetrics; HR, hazard ratio; PR, progesterone receptor; Ref, reference; WT-1, Wilms’ tumour-1. * HRs and 95% CIs were calculated with the use of the Cox proportional-hazards regression model. ** Test for interaction based on dietary total purine intake, dietary xanthine intake, dietary hypoxanthine intake, dietary adenine intake, or dietary guanine intake.

In the subgroup analyses, we found that the majority of the results were consistent with the main findings and that the protective effect of the purine intake on OC survival was slightly stronger in patients with non-serous histological subtypes, no residual lesions, and negative expressions of WT-1, PR. Similar protective effects were seen in the ER, vimentin-positive patients (Figure 2; Supplementary Tables S2 and S3). Furthermore, we found significant multiplicative interactions between residual lesions and the consumption of total purine, xanthine, hypoxanthine, adenine, and guanine; significant multiplicative interactions were further observed between the expression of WT-1 and hypoxanthine intake. Moreover, there was also a multiplicative interaction between the expression of ER and PR and the intake of xanthine and hypoxanthine, respectively (Supplementary Tables S2 and S3; Supplementary Figure S1). Additionally, additive interactions between WT-1 expression and total purine, xanthine, hypoxanthine, adenine, and guanine intake were observed (Supplementary Table S4). Immunohistochemical images of the participants with different levels of xanthine intake and hypoxanthine intake are shown in Supplementary Figure S2. The results from the sensitivity analyses were consistent with the main findings (Supplementary Tables S5–S7). Notably, we observed a curvilinear association between the total purine intake and OC survival (*p* non-linear < 0.05; Supplementary Figure S3).

## 4. Discussion

In this prospective cohort study, we first observed a significant positive association of total purine intake, especially xanthine intake, with mortality in patients with OC. Additionally, there were significant additive interactions between WT-1 and total purine intake. Furthermore, there was a multiplicative interaction between ER and PR expression and xanthine intake, and the same relationship was observed between WT-1, ER, and PR expression and hypoxanthine intake. Remarkably, we observed a non-linear association between total purine intake and OC survival.

To the best of our knowledge, no previous studies focused on the relationship between purine intake and OC prognosis. Therefore, our results provide new evidence on this topic. Our results suggest that there is a significant negative correlation between total purine intake and OC mortality. The following reasons can be given to support our findings. First, purines are mainly derived from proteins, and higher total purine intake within a reasonable range means better nutritional supplementation, which has been reported in previous studies to have a positive effect on improving the prognosis of OC [29]. Second, in an analysis of purine and OC mortality across categories, only xanthine intake was observed to have a statistically significant association with reduced OC mortality. Therefore, it can be speculated that the function of total purine in reducing OC mortality is mainly provided by xanthine. The reason for this effect may be that tea intake is very common in the dietary pattern of the Chinese population. Previous studies reported that tea is mainly rich in xanthine and other derivative substances, such a caffeine [30]. Furthermore, the intake of foods rich in xanthine, such as tea, was found to be associated with decreased OC occurrence and better prognosis [31]. For example, a case-control study in China by Min Zhang et al. included 254 patients with histologically confirmed epithelial ovarian cancer as a case group and 652 controls, including 340 hospital visitors, 261 non-cancer hospital outpatients, and 51 women recruited from the community. By collecting tea-drinking information from the participants through questionnaires and interviews, it was finally concluded that women who drink tea daily have a lower risk of OC than women who never drink tea (OR: 0.43, 95% CI = 0.30–0.63) [32]. Moreover, in a case-control study conducted in Denmark by Camilla F. et al., 267 women with ovarian cancer, 115 women with borderline ovarian tumors, and 911 randomly selected control women were surveyed for their tea and coffee consumption using a questionnaire. The results showed that the total caffeine intake from coffee and tea had a significant negative correlation with the inhibition of OC occurrence and development (OR = 0.93; 95% CI = 0.88–0.98 per 100 mg/day) [33]. Furthermore, Ann M et al. found that caffeine regulates tumor inhibition through several endogenous pathways, such as caspase-3 and p53 pathways [34]. However, the exact biological mechanism remains unclear, and further studies are needed to confirm our findings.

In addition, this may be the first study to explore the potential additive interaction between diet and genes. The results indicate that the effect of the interaction between high WT-1 protein expression and high purine intake on OC mortality is greater than the sum of the two individual effects. Given this result, it is possible to speculate as to the effect of purine intake on WT-1 protein expression. Notably, the observed additive interactions between WT-1 protein gene expression and purine intake have public-health implications, as they can be used to identify individuals who are more likely to benefit from targeted interventions that increase purine intake. In addition, we also observed some multiplicative interactions, such as the expression of ER and PR and the intake of xanthine and hypoxanthine, respectively. We inferred that the intake of xanthine and hypoxanthine has a synergistic effect with the expression of ER and PR. In other words, the intake of xanthine and hypoxanthine may enhance the effect of ER and PR on the prognosis of OC, thereby reducing the mortality of OC.

Moreover, we found a non-linear association between total purine intake and OC survival. Briefly, purines are mainly ingested through high-protein diets, and in the right range, high purine intake might indicate better dietary nutrition, which is often associated with lower OC mortality [29]. However, this does not mean that the protective effect increases infinitely with purine intake; on the contrary, after excessive purine intake, the production of a large amount of SUA in the body leads to the occurrence of hyperuricemia, which further mediates oxidative stress [35]. This negative effect might gradually counteract the antioxidant effect of SUA itself, along with the protective effect of purine on OC mortality; therefore, the trend of the increased protective effect of purines on OC mortality tends to be inconspicuous (Supplementary Figure S3). The optimal purine intake should be further explored in subsequent studies.

Several possible pathways could explain the relationship between total purine intake and improved OC survival. The P1 adenosine receptors are composed of four members (A1, A2A, A2B, and A3) [36]. Purine biosynthesizes adenosine through a biological pathway, while adenosine acts on A2A and A3 receptors to achieve immunosuppression and cell-growth regulation, thereby affecting immune-cell function, cancer growth, and metastatic spread [36]. However, some studies have shown that these receptors are antagonized by xanthine, including caffeine, and affect many cells and organs, performing a cytoprotective function [37,38]. Moreover, purine intake produces SUA in the body, which is a powerful antioxidant and acts as a scavenger of singlet oxygen and radicals by influencing the toxic and carcinogenic effects of oxygen radicals to prevent cellular oxidative stress and reduce the occurrence and development of cancer [15,16]. Furthermore, reduced cellular oxidative stress can cause HO-1, an enzyme that causes stress, to express less [39]. It has been discovered that HO-1, a new oncogene, is highly expressed in a range of gynecological malignancies, including OC, and that it is intricately linked to cancer growth, metastasis, immunological control, and angiogenesis [39]. The protective impact of purine consumption on OC survival may be explained by the interaction of these possible mechanisms. However, purine participates in the complex biological metabolic process in vivo and its potential mechanisms are still unclear. Future studies are needed to further clarify this point. It is worth noting that SUA is related to OC mortality through hormesis [40]; excessive SUA due to purine intake mediates high-SUA hematic disease, which in turn, causes tumorigenesis and transformation by increasing stress on reactive-oxygen/nitrogen-species synthesis and cyclo-oxygenase activation [35]. When this cancer-promoting effect outweighs the protective effect, on the macro level, purine intake might be associated with higher OC mortality. The results of a prospective study support this hypothesis. The study included more than 28,000 older Austrian women and reported that high SUA due to high purine intake was independently associated with an increased risk of total cancer mortality (HR = 1.27, 95% CI = 1.08–1.48) [40].

Our investigation has several advantages. To our knowledge, this is the only epidemiological study to explore the relationship between purine intake and mortality in OC patients. Second, this study has a prospective design, a high baseline-survey participation rate (93%), and a high survey follow-up rate (above 90%), which might have reduced follow-up and selection biases. In addition, we extensively collected potential confounding factors and adjusted them during the analysis. These might have reduced the interference of confounding factors and increase the reliability of the results. Moreover, we performed multifaceted subgroup analyses and multivariate analyses to strengthen the reliability of the primary results.

However, it is necessary to discuss the potential limitations of this study. Firstly, because our study sample was drawn exclusively from China, whose population has different characteristics from those of high-income countries, extrapolation to other populations may be limited. Secondly, residual confounding from imprecise measures of the included covariates, such as dietary information, including total purine and different kinds of purine intake, which may be more liable to misclassification, cannot be excluded. However, we used high-reliability and high-validity questionnaires and collected the information with trained staff using the face-to-face interview method, which has a reasonable degree of credibility. Therefore, our method may have helped us to reduce misclassification and information bias. Thirdly, the current study only collected dietary intake prior to diagnosis, and some of the participants may have changed their dietary habits during the follow-up period. However, information has been obtained that suggests a strong correlation between pre- and post-diagnostic diets [41], and in our study, only a tiny minority of the patients (23.9%) modified their dietary habits. Furthermore, the method of OC treatment may influence OC prognosis. In our analysis, however, over 95% of the OC patients received surgery as well as paclitaxel-and-platinum combination chemotherapy. Therefore, the final results of this study were not affected.

## 5. Conclusions

In conclusion, this prospective cohort analysis found an inverse relationship between pre-diagnosis dietary purine intake, particularly xanthine intake, and OC mortality. Further clarification of these findings is warranted.

## Figures and Tables

**Table 1:** Baseline characteristics of 703 patients with ovarian cancer by tercile of dietary total purine intake in the OOPS.

**Characteristics**	**All Patients**	**Terciles of Total Purine Intake**			***p* Value**
		**I (*n* = 234)**	**II (*n* = 234)**	**III (*n* = 235)**	
Range (mg/day)		<179.48	179.48–<189.05	≥189.05	
Median (IQR) age at diagnosis (years)	53.00 (12.00)	53.00 (12.00)	53.00 (12.00)	54.00 (13.00)	0.98
Median (IQR) follow-up time (months)	31.20 (26.84)	31.28 (24.73)	29.90 (26.00)	33.67 (27.67)	0.14
Median (IQR) body-mass index (kg/m^2^)	23.30 (4.20)	23.30 (3.60)	22.90 (4.20)	23.30 (5.00)	0.19
Median (IQR) physical activity (MET h/d)	14.10 (15.70)	14.60 (15.50)	12.55 (15.40)	14.70 (15.70)	0.33
Ever smoked cigarettes	68 (9.67)	24 (10.26)	23 (9.83)	21 (8.94)	0.89
Ever consumed alcohol	149 (21.19)	38 (16.24)	56 (23.93)	55 (23.40)	0.08
Ever experienced menopause	508 (72.26)	167 (71.37)	168 (71.79)	173 (73.62)	0.85
Parity					<0.05
≤1	505 (71.83)	154 (65.81)	175 (74.79)	176 (74.89)	
≥2	198 (28.17)	80 (34.19)	59 (25.21)	59 (25.11)	
Educational level					0.11
Junior secondary or below	375 (53.34)	138 (58.97)	120 (51.28)	117 (49.79)	
Senior high school/technical secondary school	147 (20.91)	44 (18.81)	57 (24.36)	46 (19.57)	
Junior college/university or above	181 (25.75)	52 (22.22)	57 (24.36)	72 (30.64)	
Income per month (CNY)					0.06
<5000	421 (59.89)	157 (67.09)	133 (56.84)	131 (55.74)	
5000 to <10000	194 (27.60)	49 (20.94)	70 (29.91)	75 (31.92)	
≥10000	88 (12.51)	28 (11.97)	31 (13.25)	29 (12.34)	
Ever changed diet					0.12
No	535 (76.10)	189 (80.77)	172 (73.50)	174 (74.04)	
Yes	168 (23.90)	45 (19.23)	62 (26.50)	61 (25.96)	
Mean (SD) total energy intake (kcal/d)	1455.75 (552.64)	1028.14 (282.69)	1368.28 (332.36)	1968.65 (521.53)	<0.05
Mean (SD) total fatty-acid intake (g/d)	23.81 (13.94)	12.78 (5.23)	21.69 (7.78)	36.90 (14.05)	<0.05
Mean (SD) cholesterol intake (mg/d)	355.75 (217.67)	215.69 (158.40)	330.71 (164.44)	520.15 (206.99)	<0.05
Mean (SD) total purine intake (mg/d)	260.26 (140.67)	129.83 (34.66)	231.54 (31.34)	418.73 (118.87)	<0.05
Mean (SD) xanthine intake (mg/d)	14.77 (8.49)	7.65 (3.04)	13.68 (4.35)	22.97 (8.30)	<0.05
Mean (SD) hypoxanthine (mg/d)	62.87 (42.80)	29.22 (14.62)	56.05 (21.53)	103.19 (44.82)	<0.05
Mean (SD) adenine intake (mg/d)	90.42 (49.63)	46.19 (13.38)	80.33 (15.47)	144.53 (44.43)	<0.05
Mean (SD) guanine intake (mg/d)	92.13 (50.92)	46.75 (13.86)	81.45 (14.56)	147.94 (45.56)	<0.05

d, day; IQR, inter-quartile range; MET, metabolic equivalents of task; OOPS, the ovarian-cancer follow-up study; SD, standard deviation. Values are numbers (percentages) unless stated otherwise.

**Table 2:** Adjusted hazard ratios (HRs) and 95% confidence intervals (CIs) for the association of dietary purine intake with mortality in 703 ovarian cancer patients *.

Characteristics		Deaths (% of Total)	Model 1	Model 2	Model 3
Total Purine (mg/day)	T1 (<179.78)	47 (36.15)	1.00 (Ref)	1.00 (Ref)	1.00 (Ref)
	T2 (179.78–<289.05)	51 (39.23)	1.08 (0.73–1.61)	1.03 (0.68–1.56)	0.88 (0.56–1.37)
	T3 (≥289.05)	32 (24.62)	0.63 (0.40–0.99)	0.67 (0.42–1.08)	0.39 (0.19–0.80)
	Continuous (per SD)	130 (100.00)	0.89 (0.75–1.07)	0.90 (0.75–1.08)	0.60 (0.41–0.88)
	*p* for trend **		<0.05	0.08	<0.05
Xanthine (mg/day)	T1 (<9.69)	51 (39.23)	1.00 (Ref)	1.00 (Ref)	1.00 (Ref)
	T2 (9.69–<16.82)	41 (31.54)	0.75 (0.50–1.13)	0.84 (0.55–1.28)	0.80 (0.51–1.25)
	T3 (≥16.82)	38 (29.23)	0.67 (0.44–1.02)	0.68 (0.44–1.05)	0.52 (0.29–0.94)
	Continuous (per SD)	130 (100.00)	0.91 (0.76–1.09)	0.91 (0.76–1.09)	0.80 (0.60–1.06)
	*p* for trend **		0.07	0.09	<0.05
Hypoxanthine (mg/day)	T1 (<38.35)	49 (37.69)	1.00 (Ref)	1.00 (Ref)	1.00 (Ref)
	T2 (38.35–<71.43)	45 (34.62)	0.88 (0.59–1.33)	0.94 (0.62–1.42)	0.90 (0.58–1.39)
	T3 (≥71.43)	36 (27.69)	0.67 (0.43–1.03)	0.71 (0.45–1.10)	0.59 (0.33–1.06)
	Continuous (per SD)	130 (100.00)	0.87 (0.73–1.05)	0.89 (0.74–1.06)	0.82 (0.63–1.07)
	*p* for trend **		0.06	0.12	0.07
Adenine (mg/day)	T1 (<61.60)	45 (34.62)	1.00 (Ref)	1.00 (Ref)	1.00 (Ref)
	T2 (61.60–<100.11)	50 (38.46)	1.11 (0.74–1.67)	1.16 (0.77–1.75)	1.08 (0.69–1.69)
	T3 (≥100.11)	35 (26.92)	0.76 (0.49–1.18)	0.80 (0.51–1.27)	0.54 (0.27–1.06)
	Continuous (per SD)	130 (100.00)	0.91 (0.76–1.09)	0.92 (0.77–1.10)	0.60 (0.41–0.87)
	*p* for trend **		0.17	0.27	0.06
Guanine (mg/day)	T1 (<63.59)	44 (33.85)	1.00 (Ref)	1.00 (Ref)	1.00 (Ref)
	T2 (63.59–<101.59)	51 (39.23)	1.16 (0.78–1.74)	1.13 (0.75–1.71)	1.06 (0.67–1.65)
	T3 (≥101.59)	35 (26.92)	0.77 (0.49–1.19)	0.79 (0.50–1.25)	0.54 (0.27–1.08)
	Continuous (per SD)	130 (100.00)	0.91 (0.76–1.09)	0.92 (0.77–1.11)	0.64 (0.44–0.92)
	*p* for trend **		0.18	0.26	0.08

CI, confidence interval; HR, hazard ratio; Ref, reference; SD, standard deviation; T, tercile. * HRs and 95% CIs were calculated with the use of the Cox proportional hazards regression model. ** Tests for trend were based on variables containing the median value for each tercile. SD of total purine: 140.67 mg/day, SD of xanthine: 8.49 mg/day, SD of hypoxanthine: 42.80 mg/day, SD of adenine: 49.63 mg/day, SD of guanine: 50.92 mg/day. Model 1 was adjusted for age at diagnosis. Model 2 was the same as Model 1 and further adjusted for education level, income level, smoke status, alcohol intake, body-mass index, physical activity, comorbidities, FIGO stage, histological type, histopathologic grade, residual lesions, menopausal status, oral contraceptive status, and parity. Model 3 was the same as Model 2 and further adjusted for dietary change and total energy, total fatty acid, and cholesterol intake.

## Data Availability

The datasets used and/or analyzed during the current study are available from the corresponding author on reasonable request.

## Supplementary Materials

The following supporting information can be downloaded at: https://www.mdpi.com/article/10.3390/nu15040931/s1, Figure S1: Kaplan-Meier survival curves for A: total purine intake; B: dietary xanthine intake; C: dietary hypoxanthine intake; D: dietary adenine intake; E: dietary guanine intake; Figure S2: Immunohistochemical images of different xanthine intake and hypoxanthine intake levels; Figure S3: HR and 95% CI of mortality among ovarian cancer patients by A: total purine intake; B: dietary xanthine intake; C: dietary hypoxanthine intake; D: dietary adenine intake; E: dietary guanine intake; Table S1: Selected clinical characteristics and associations with mortality among 703 ovarian cancer patients; Table S2: Subgroup analyses of demographical and clinical characteristics for adjusted hazard ratio (HR) and 95% confidence interval (CI) for the association of dietary purine intake with mortality of ovarian cancer patients; Table S3: Subgroup analyses of demographical and clinical characteristics for adjusted hazard ratio (HR) and 95% confidence interval (CI) for the association of dietary purine intake with mortality of ovarian cancer patients; Table S4: Relative excess risk due to interaction (RERI) and 95% confidence interval (CI) * of survival by the purine intake among ovarian cancer patients; Table S5: Adjusted hazard ratio (HR) and 95% confidence interval (CI) * of survival by the tertiles of purine intake among ovarian cancer patients: excluding deaths occurring in one year of follow-up; Table S6: Adjusted hazard ratio (HR) and 95% confidence interval (CI) * of survival by the tertiles of purine intake among ovarian cancer patients: using the residual method and energy density method; Table S7: Adjusted hazard ratio (HR) and 95% confidence interval (CI) * of survival by the tertiles of purine intake among ovarian cancer patients: excluding dietary change of follow-up.
