# Supplementary material for: Purine Intake and All-Cause Mortality in Ovarian Cancer: Results from a Prospective Cohort Study
Source: Nutrients. 2023 Feb 13;15(4):931. doi: 10.3390/nu15040931 (PMC9965699; doi:10.3390/nu15040931)
Supplement: Supplementary file 1 [file nutrients-15-00931-s001.zip › nutrients-2110373-supplementary materials.pdf]

eMethods. Detailed data collection, follow-up and outcome information, and statistical analysis of specific methods.

a. Data collection

Standard self-administered questionnaires collected the sociodemographic information and lifestyle of patients with OC including education, income, diet, physical activity, alcohol use (at least once a week for more than six months), smoking (at least one cigarette per day for more than six months), and reproductive factors. Moreover, the clinical information was taken from the electronic medical records of the Shengjing hospital information system, including age at diagnosis, histological type (serous and non-serous), histopathologic grade (well, moderately, and poorly differentiated), International Federation of Gynecology and Obstetrics (FIGO) stage (I-II, III-IV, and unknown), residual lesions (none, <1, and ⩾1 cm), and comorbidities (yes and no).

b. Follow-up and outcome

The outcome was all-cause mortality in the current analysis. Data extracted from medical records every six months, record data linked to the Liaoning Providence Center for Disease Control and Prevention, and active follow-up were applied to determine participants' vital status. From the date of pathologic OC diagnosis to the date of death from any cause, or, for patients who were still alive, the date of last follow-up, the survival time was determined (March 31, 2021).

c. Statistical analysis

We used the Kaplan–Meier technique to plot a crude survival curve and estimate crude mortality probabilities. The Cox proportional hazards regression model was used to assess the association of pre-diagnosis total purine intake with mortality by calculating the hazard ratio (HR) and 95% confidence interval (CI). The proportional hazards assumption was estimated by adding an interaction term between each activity variable and log survival time, and all variables satisfied the conditions (all P>0.05). In this study, participants were divided into tertiles based on purine intake, including total purine, xanthine, hypoxanthine, adenine, and guanine, with the lowest tertile serving as the reference group. We estimated the P trend by including the median in each tertile as a continuous variable in the Cox regression models.

Several potential confounders were adjusted. In the first model, we adjusted for age at diagnosis (< 50 or ≥ 50 years). In the second model, we further included various lifestyle-related covariates and clinical relative factors, including education (junior secondary or below, senior high school/technical secondary school, and junior college/university or above), monthly household income (<5, 5-10, and ≥10 RMB; thousand yuan), menopausal status (yes or no), oral contraceptive status (yes or no), smoke status (yes or no), drink status (yes or no), physical activity [continuous, metabolic equivalent task (MET)/hours/days], BMI (continuous, kg/m2), parity (≤ 1 or ≥ 2 times), comorbidities (yes or no), histopathologic grade (well, moderately, or poorly differentiated), histological type (serous or non-serous), FIGO stage (I-II, III-IV, or unknown), and residual lesions (none, <1 or ≥ 1 cm). In the third model, we further adjusted for dietary change (yes or no), total energy (continuous, kcal/d), total fatty acid intake (continuous, g/d), and cholesterol intake (continuous, mg/d) based on the second model.

Additionally, stratified exploratory analyses were performed by age at diagnosis (< 50 or ≥ 50 years), menopausal status (yes or no), BMI (< 25 or ≥ 25 kg/m2), histological type (serous or non-serous), FIGO stage (I-II or III-IV), residual lesions (no or yes), WT-1 (positive or negative), ER (positive or negative), PR (positive or negative), and Vimentin (positive or negative).

Supplementary Table S1. Selected clinical characteristics and associations with mortality among 703 ovarian cancer patients.

Characteristics	No. of deaths/total (%)	Adjusted HR * (95%CI)
**Age at diagnosis (years)**		
≤ 50	45/258 (17.44)	1.00 (Ref)
> 50	85/445 (19.10)	1.15 (0.70-1.87)
**Histological type**		
Serous	92/479 (19.21)	1.00 (Ref)
Non-serous	38/224 (16.96)	1.03 (0.51-2.08)
**Histopathologic grade**		
Well differentiated	5/56 (8.93)	1.00 (Ref)
Moderately differentiated	7/48 (14.58)	1.56 (0.36-6.71)
Poorly differentiated	118/599 (19.70)	2.20 (0.43-11.26)
**FIGO stage**		
I-II	41/342 (11.99)	1.00 (Ref)
III-IV	89/338 (26.33)	2.98 (1.67-5.30)
**Residual lesions**		
No	82/553 (14.83)	1.00 (Ref)
< 1 cm	31/106 (29.25)	2.04 (1.09-3.82)
≥ 1 cm	17/44 (38.64)	3.93 (1.94-7.95)
**Comorbidities**		
No	74/393 (18.83)	1.00 (Ref)
Yes	56/310 (18.06)	0.78 (0.49-1.24)

**Wilms' tumour-1**		
Negative	44/190 (23.16)	1.00 (Ref)
Positive	59/378 (15.61)	0.46 (0.26-0.84)
**Estrogen Receptor**		
Negative	30/129 (23.26)	1.00 (Ref)
Positive	81/454 (17.84)	0.52 (0.26-1.04)
**Progesterone Receptor**		
Negative	61/262 (23.28)	1.00 (Ref)
Positive	50/321 (15.58)	0.72 (0.42-1.24)
**Vimentin**		
Negative	65/359 (18.11)	1.00 (Ref)
Positive	29/156 (18.59)	1.03 (0.58-1.83)

CI, confidence interval; FIGO, The International Federation of Gynecology and Obstetrics; HR, hazard ratio; Ref, reference; WT-1, Wilms' tumour-1.

* Mutually adjusted for all other variables listed in the table.

Supplementary Table S2. Subgroup analyses of demographical and clinical characteristics for adjusted hazard ratio (HR) and 95% confidence interval (CI) for the association of dietary purine intake with mortality of ovarian cancer patients

Characteristics	Tertiles of dietary total purine intake				Tertiles of dietary xanthine intake				Tertiles of dietary hypoxanthine intake			
	I	II	III	$P_{interaction}$*	I	II	III	$P_{interaction}$*	I	II	III	$P_{interaction}$*
**Age at diagnosis (years)**				0.44				0.47				0.87
≤ 50	1.00 (Ref)	1.05 (0.46-2.39)	0.95 (0.26-3.50)		1.00 (Ref)	1.24 (0.56-2.76)	0.63 (0.20-1.94)		1.00 (Ref)	0.52 (0.20-1.30)	0.23 (0.06-0.93)	
> 50	1.00 (Ref)	0.70 (0.40-1.22)	0.20 (0.08-0.50)		1.00 (Ref)	0.50 (0.28-0.90)	0.33 (0.16-0.70)		1.00 (Ref)	0.92 (0.53-1.60)	0.59 (0.29-1.17)	
**Menopausal status**				0.91				0.62				0.45
Yes	1.00 (Ref)	0.87 (0.51-1.48)	0.31 (0.13-0.72)		1.00 (Ref)	0.76 (0.45-1.30)	0.50 (0.25-1.00)		1.00 (Ref)	1.11 (0.66-1.88)	0.59 (0.30-1.16)	
No	1.00 (Ref)	0.92 (0.35-2.40)	0.43 (0.08-2.25)		1.00 (Ref)	1.05 (0.41-2.69)	0.27 (0.07-1.07)		1.00 (Ref)	0.34 (0.12-1.00)	0.23 (0.05-1.17)	
**Body mass index (kg/m^2)**				0.58				0.51				0.92
< 25	1.00 (Ref)	0.97 (0.59-1.59)	0.40 (0.17-0.93)		1.00 (Ref)	0.81 (0.48-1.35)	0.51 (0.27-1.00)		1.00 (Ref)	0.79 (0.48-1.31)	0.48 (0.24-0.95)	
≥ 25	1.00 (Ref)	0.67 (0.22-2.02)	0.43 (0.10-1.96)		1.00 (Ref)	0.75 (0.26-2.16)	0.65 (0.15-2.78)		1.00 (Ref)	1.04 (0.35-3.14)	1.24 (0.37-4.14)	
**Histological type**				0.33				0.77				0.54
Serous	1.00 (Ref)	0.83 (0.48-1.42)	0.51 (0.22-1.17)		1.00 (Ref)	0.71 (0.42-1.21)	0.52 (0.26-1.05)		1.00 (Ref)	0.89 (0.52-1.50)	0.59 (0.30-1.18)	
Non-serous	1.00 (Ref)	0.82 (0.32-2.10)	0.08 (0.01-0.49)		1.00 (Ref)	1.31 (0.51-3.37)	0.38 (0.11-1.31)		1.00 (Ref)	0.70 (0.29-1.68)	0.32 (0.10-1.07)	
**FIGO stage**				0.80				0.14				0.75
I-II	1.00 (Ref)	0.79 (0.34-1.83)	0.32 (0.08-1.29)		1.00 (Ref)	0.57 (0.25-1.31)	0.17 (0.05-0.55)		1.00 (Ref)	1.22 (0.55-2.71)	0.64 (0.21-1.94)	
III-IV	1.00 (Ref)	1.01 (0.58-1.77)	0.48 (0.20-1.15)		1.00 (Ref)	0.97 (0.55-1.70)	0.77 (0.38-1.57)		1.00 (Ref)	0.69 (0.40-1.20)	0.52 (0.25-1.05)	
**Residual lesions**				< 0.05				< 0.05				< 0.05
No	1.00 (Ref)	0.80 (0.46-1.40)	0.29 (0.12-0.69)		1.00 (Ref)	0.85 (0.49-1.49)	0.48 (0.23-1.00)		1.00 (Ref)	0.77 (0.45-1.32)	0.47 (0.22-0.97)	

Yes	1.00 (Ref)	0.93 (0.40-2.14)	0.55 (0.14-2.21)		1.00 (Ref)	0.97 (0.41-2.30)	0.48 (0.16-1.42)		1.00 (Ref)	1.21 (0.55-2.66)	0.72 (0.26-2.04)	
**Wilms' tumour-1**				0.13				0.09				< 0.05
Positive	1.00 (Ref)	0.95 (0.45-2.00)	0.93 (0.35-2.52)		1.00 (Ref)	0.53 (0.25-1.14)	0.84 (0.36-1.92)		1.00 (Ref)	0.78 (0.39-1.58)	0.63 (0.27-1.48)	
Negative	1.00 (Ref)	0.47 (0.19-1.67)	0.02 (0.00-0.18)		1.00 (Ref)	0.94 (0.41-2.13)	0.03 (0.00-0.17)		1.00 (Ref)	1.19 (0.49-2.89)	0.75 (0.21-2.63)	
**Estrogen Receptor**				0.05				< 0.05				< 0.05
Positive	1.00 (Ref)	0.53 (0.29-0.98)	0.37 (0.15-0.93)		1.00 (Ref)	0.54 (0.29-0.99)	0.44 (0.21-0.94)		1.00 (Ref)	0.86 (0.49-1.53)	0.50 (0.24-1.08)	
Negative	1.00 (Ref)	1.74 (0.53-5.77)	0.12 (0.01-1.13)		1.00 (Ref)	2.03 (0.59-7.00)	0.11 (0.01-0.91)		1.00 (Ref)	0.98 (0.33-2.89)	0.75 (0.15-3.82)	
**Progesterone Receptor**				0.05				< 0.05				< 0.05
Positive	1.00 (Ref)	0.58 (0.27-1.24)	0.31 (0.09-1.04)		1.00 (Ref)	0.52 (0.23-1.14)	0.31 (0.11-0.87)		1.00 (Ref)	0.81 (0.37-1.76)	0.46 (0.17-1.34)	
Negative	1.00 (Ref)	1.11 (0.56-2.21)	0.26 (0.09-0.78)		1.00 (Ref)	1.09 (0.56-2.13)	0.46 (0.18-1.23)		1.00 (Ref)	0.93 (0.47-1.83)	0.56 (0.22-1.42)	
**Vimentin**				0.90				0.98				0.72
Positive	1.00 (Ref)	0.33 (0.09-1.21)	0.02 (0.00-0.28)		1.00 (Ref)	0.71 (0.22-2.29)	0.23 (0.03-1.70)		1.00 (Ref)	0.79 (0.21-2.92)	0.21 (0.03-1.26)	
Negative	1.00 (Ref)	1.01 (0.51-2.01)	0.48 (0.17-1.42)		1.00 (Ref)	1.04 (0.53-2.03)	0.55 (0.22-1.36)		1.00 (Ref)	0.85 (0.45-1.61)	0.50 (0.21-1.17)	

CI, confidence interval; FIGO, The International Federation of Gynecology and Obstetrics; HR, hazard ratio; Ref, reference.

*Test for interaction based on dietary total purine intake, dietary xanthine intake, or dietary hypoxanthine intake.

HR and 95% CI were calculated through the Cox proportional hazards regression model with adjustment for age at diagnosis, education level, income level, smoke status, alcohol intake, comorbidities, body mass index, physical activity, FIGO stage, histological type, histopathologic grade, menopausal status, oral contraceptive status, parity, residual lesions, total energy intake, dietary change, total fatty acid intake, and cholesterol intake.

Supplementary Table S3. Subgroup analyses of demographical and clinical characteristics for adjusted hazard ratio (HR) and 95% confidence interval (CI) for the association of dietary purine intake with mortality of ovarian cancer patients

Characteristics	Tertiles of dietary adenine intake				Tertiles of dietary guanine intake			
	I	II	III	$P_{interaction}$*	I	II	III	$P_{interaction}$*
**Age at diagnosis (years)**				0.22				0.19
≤ 50	1.00 (Ref)	1.18 (0.51-2.77)	1.45 (0.50-4.24)		1.00 (Ref)	1.20 (0.52-2.77)	1.66 (0.59-4.64)	
> 50	1.00 (Ref)	0.85 (0.50-1.46)	0.20 (0.08-0.52)		1.00 (Ref)	0.86 (0.50-1.48)	0.18 (0.07-0.46)	
**Menopausal status**				0.52				0.56
Yes	1.00 (Ref)	1.15 (0.68-1.95)	0.44 (0.19-1.02)		1.00 (Ref)	1.10 (0.65-1.88)	0.43 (0.19-0.99)	
No	1.00 (Ref)	1.11 (0.42-2.91)	0.64 (0.16-2.60)		1.00 (Ref)	1.30 (0.51-3.30)	0.69 (0.17-2.85)	
**Body mass index (kg/m^2)**				0.30				0.21
< 25	1.00 (Ref)	1.15 (0.70-1.90)	0.65 (0.30-1.43)		1.00 (Ref)	1.10 (0.67-1.82)	0.76 (0.35-1.66)	
≥ 25	1.00 (Ref)	0.95 (0.32-2.83)	0.60 (0.11-3.21)		1.00 (Ref)	0.95 (0.32-2.81)	0.27 (0.05-1.54)	
**Histological type**				0.28				0.29
Serous	1.00 (Ref)	1.07 (0.63-1.83)	0.80 (0.35-1.83)		1.00 (Ref)	1.18 (0.68-2.03)	0.74 (0.32-1.70)	
Non-serous	1.00 (Ref)	1.13 (0.46-2.76)	0.13 (0.02-0.65)		1.00 (Ref)	0.88 (0.35-2.20)	0.16 (0.04-0.76)	
**FIGO stage**				0.76				0.94
I-II	1.00 (Ref)	0.98 (0.44-2.20)	0.32 (0.08-1.28)		1.00 (Ref)	0.86 (0.38-1.93)	0.42 (0.11-1.54)	
III-IV	1.00 (Ref)	1.23 (0.71-2.14)	0.65 (0.28-1.52)		1.00 (Ref)	1.32 (0.75-2.31)	0.59 (0.25-1.39)	
**Residual lesions**				< 0.05				< 0.05
No	1.00 (Ref)	1.18 (0.68-2.05)	0.45 (0.18-1.08)		1.00 (Ref)	1.03 (0.59-1.78)	0.37 (0.15-0.90)	

Yes	1.00 (Ref)	0.83 (0.36-1.91)	0.42 (0.12-1.50)		1.00 (Ref)	1.02 (0.45-2.33)	0.66 (0.18-2.34)	
**Wilms' tumour-1**				0.10				0.08
Positive	1.00 (Ref)	1.16 (0.57-2.37)	1.12 (0.40-3.08)		1.00 (Ref)	1.65 (0.79-3.47)	1.20 (0.42-3.40)	
Negative	1.00 (Ref)	0.81 (0.35-1.88)	0.04 (0.01-0.28)		1.00 (Ref)	0.78 (0.34-1.81)	0.06 (0.01-0.35)	
**Estrogen Receptor**				0.07				0.08
Positive	1.00 (Ref)	0.69 (0.39-1.24)	0.56 (0.23-1.37)		1.00 (Ref)	0.81 (0.45-1.46)	0.54 (0.22-1.33)	
Negative	1.00 (Ref)	1.72 (0.51-5.86)	0.05 (0.01-0.57)		1.00 (Ref)	0.78 (0.27-2.28)	0.05 (0.01-0.51)	
**Progesterone Receptor**				0.16				0.12
Positive	1.00 (Ref)	0.90 (0.41-1.95)	0.60 (0.19-1.91)		1.00 (Ref)	1.02 (0.47-2.23)	0.59 (0.19-1.88)	
Negative	1.00 (Ref)	0.84 (0.44-1.63)	0.16 (0.05-0.50)		1.00 (Ref)	0.81 (0.41-1.57)	0.18 (0.06-0.56)	
**Vimentin**				0.89				0.96
Positive	1.00 (Ref)	0.25 (0.07-0.86)	0.01 (0.00-0.19)		1.00 (Ref)	0.61 (0.16-2.25)	0.01 (0.00-0.18)	
Negative	1.00 (Ref)	1.23 (0.64-2.39)	0.58 (0.21-1.57)		1.00 (Ref)	0.81 (0.41-1.61)	0.58 (0.21-1.64)	

CI, confidence interval; FIGO, The International Federation of Gynecology and Obstetrics; HR, hazard ratio; Ref, reference.

*Test for interaction based on dietary adenine intake, or dietary guanine intake.

HR and 95% CI were calculated through the Cox proportional hazards regression model with adjustment for age at diagnosis, education level, income level, smoke status, alcohol intake, comorbidities, body mass index, physical activity, FIGO stage, histological type, histopathologic grade, menopausal status, oral contraceptive status, parity, residual lesions, total energy intake, dietary change, total fatty acid intake, and cholesterol intake.

Supplementary Table S4. Relative excess risk due to interaction (RERI) and 95% confidence interval (CI) * of survival by the purine intake among ovarian cancer patients

Characteristics	Purine Intake RERI * (95% CI)				
	Total Purine	Xanthine	Hypoxanthine	Adenine	Guanine
**Wilms' tumour-1**	**0.62 (0.36-0.88)**	**0.69 (0.47-0.91)**	**0.56 (0.26-0.86)**	**0.56 (0.26-0.86)**	**0.71 (0.51-0.92)**
**Estrogen Receptor**	0.42 (-0.06-0.90)	0.38 (-0.16-0.92)	0.33 (-0.25-0.90)	0.37 (-0.14-0.88)	**0.56 (0.19-0.93)**
**Progesterone Receptor**	0.18 (-0.39-0.75)	0.26 (-0.29-0.80)	0.19 (-0.39-0.78)	0.12 (-0.46-0.71)	0.21 (-0.35-0.78)
**Vimentin**	-1.96 (-6.10-2.19)	-0.87 (-3.32-1.59)	-0.48 (-2.47-1.50)	-1.03 (-3.71-1.65)	-1.67 (-5.37-2.02)

CI, confidence interval; RERI, relative excess risk due to interaction; HR, hazard ratio; Ref, reference.

* Adjustment for age at diagnosis, education level, income level, smoke status, alcohol intake, comorbidities, body mass index, physical activity, FIGO stage, histological type, histopathologic grade, menopausal status, oral contraceptive status, parity, residual lesions, total energy intake, dietary change, total fatty acid intake, and cholesterol intake.

Supplementary Table S5. Adjusted hazard ratio (HR) and 95% confidence interval (CI) * of survival by the tertiles of purine intake among ovarian cancer patients: excluding deaths occurring in one year of follow-up

**Characteristics**		**Adjusted HR * (95%CI)**
**Total Purine (mg/day)**	T1(< 178.75)	1.00 (Ref)
	T2 (178.75 -< 290.57)	0.81 (0.47-1.41)
	T3 (≥ 290.57)	0.40 (0.17-0.95)
	P for trend **	< 0.05
**Xanthine (mg/day)**	T1(< 9.69)	1.00 (Ref)
	T2 (9.69 -< 16.91)	0.49 (0.28-0.87)
	T3 (≥ 16.91)	0.52 (0.27-1.02)
	P for trend **	0.07
**Hypoxanthine (mg/day)**	T1(< 38.35)	1.00 (Ref)
	T2 (38.35 -< 71.40)	0.80 (0.47-1.37)
	T3 (≥ 71.40)	0.59 (0.26-1.06)
	P for trend **	0.07
**Adenine (mg/day)**	T1(< 61.13)	1.00 (Ref)
	T2 (61.13 -< 100.11)	1.21 (0.70-2.08)
	T3 (≥ 100.11)	0.63 (0.28-1.43)
	P for trend **	0.23
**Guanine (mg/day)**	T1(< 63.11)	1.00 (Ref)
	T2 (63.11 -< 102.75)	0.97 (0.56-1.67)
	T3 (≥ 102.75)	0.54 (0.27-1.08)
	P for trend **	0.08

CI, confidence interval; HR, hazard ratio; Ref, reference; T, tertile.

* HR and 95% CI were calculated through the Cox proportional hazards regression model with adjustment for age at diagnosis, education level, income level, smoke status, alcohol intake, comorbidities, body mass index, physical activity, FIGO stage, histological type, histopathologic grade, menopausal status, oral contraceptive status, parity, residual lesions, total energy intake, dietary change, total fatty acid intake, and cholesterol intake.

** Tests for trend were based on variables containing the median value for each tertile.

Supplementary Table S6. Adjusted hazard ratio (HR) and 95% confidence interval (CI) * of survival by the tertiles of purine intake among ovarian cancer patients: using the residual method and energy density method

Characteristics	Adjustment by the residual method		Adjustment by energy density method	
	**Range (mg/d)**	**Adjusted HR * (95%CI)**	**Range (mg/1000kcal)**	**Adjusted HR * (95%CI)**
**Total Purine**	T1(< 221.82)	1.00 (Ref)	T1(< 144.64)	1.00 (Ref)
	T2 (221.82 -< 286.97)	0.66 (0.42-1.02)	T2 (144.64 -< 226.78)	0.67 (0.42-1.05)
	T3 (≥ 286.97)	0.56 (0.34-0.92)	T3 (≥ 226.78)	0.52 (0.31-0.88)
	P for trend **	< 0.05	*P* for trend **	< 0.05
**Xanthine**	T1(< 12.11)	1.00 (Ref)	T1(< 7.91)	1.00 (Ref)
	T2 (12.11 -< 16.41)	0.63 (0.40-1.00)	T2 (7.91 -< 11.30)	0.58 (0.37-0.92)
	T3 (≥ 16.41)	0.71 (0.46-1.10)	T3 (≥ 11.30)	0.67 (0.43-1.05)
	P for trend **	0.18	*P* for trend **	0.12
**Hypoxanthine**	T1(< 47.35)	1.00 (Ref)	T1(< 30.57)	1.00 (Ref)
	T2 (47.35 -< 70.76)	0.90 (0.58-1.41)	T2 (30.57 -< 64.45)	0.74 (0.47-1.15)
	T3 (≥ 70.76)	0.64 (0.39-1.06)	T3 (≥ 64.45)	0.49 (0.29-0.82)
	P for trend **	0.07	*P* for trend **	< 0.05
**Adenine**	T1(< 77.05)	1.00 (Ref)	T1(< 50.43)	1.00 (Ref)
	T2 (77.05 -< 98.67)	0.61 (0.39-0.95)	T2 (50.43 -< 67.21)	0.68 (0.44-1.07)
	T3 (≥ 98.67)	0.48 (0.29-0.79)	T3 (≥ 67.21)	0.43 (0.25-0.74)
	P for trend **	< 0.05	*P* for trend **	< 0.05
**Guanine**	T1(< 77.48)	1.00 (Ref)	T1(< 51.88)	1.00 (Ref)
	T2 (77.48 -< 100.47)	0.76 (0.49-1.17)	T2 (51.88 -< 69.39)	0.71 (0.46-1.11)
	T3 (≥ 100.74)	0.59 (0.36-0.95)	T3 (≥ 69.39)	0.48 (0.28-0.81)
	P for trend **	< 0.05	*P* for trend **	< 0.05

CI, confidence interval; HR, hazard ratio; Ref, reference; T, tertile.

* HR and 95% CI were calculated through the Cox proportional hazards regression model with adjustment for age at diagnosis, education level, income level, smoke status, alcohol intake, comorbidities, body mass index, physical activity, FIGO stage, histological type, histopathologic grade, menopausal status, oral contraceptive status, parity, residual lesions, total energy intake, dietary change, total fatty acid intake, and cholesterol intake.

** Tests for trend were based on variables containing the median value for each tertile.

Supplementary Table S7. Adjusted hazard ratio (HR) and 95% confidence interval (CI) * of survival by the tertiles of purine intake among ovarian cancer patients: excluding dietary change of follow-up

Characteristics		Adjusted HR * (95%CI)
**Total Purine (mg/day)**	T1(< 175.33)	1.00 (Ref)
	T2 (175.33 -< 286.76)	0.95 (0.57-1.57)
	T3 (≥ 286.76)	0.39 (0.17-0.89)
	P for trend **	< 0.05
**Xanthine (mg/day)**	T1(< 9.52)	1.00 (Ref)
	T2 (9.52 -< 16.49)	0.70 (0.42-1.17)
	T3 (≥ 16.49)	0.46 (0.24-0.91)
	P for trend **	< 0.05
**Hypoxanthine (mg/day)**	T1(< 38.06)	1.00 (Ref)
	T2 (38.06 -< 70.98)	0.83 (0.50-1.39)
	T3 (≥ 70.98)	0.59 (0.30-1.15)
	P for trend **	0.12
**Adenine (mg/day)**	T1(< 59.74)	1.00 (Ref)
	T2 (59.74 -< 98.67)	1.32 (0.78-2.23)
	T3 (≥ 98.67)	0.75 (0.36-1.58)
	P for trend **	0.36
**Guanine (mg/day)**	T1(< 61.46)	1.00 (Ref)
	T2 (61.46 -< 100.44)	1.36 (0.82-2.27)
	T3 (≥ 100.44)	0.60 (0.28-1.29)
	P for trend **	0.16

CI, confidence interval; HR, hazard ratio; Ref, reference; T, tertile.

* HR and 95% CI were calculated through the Cox proportional hazards regression model with adjustment for age at diagnosis, education level, income level, smoke status, alcohol intake, comorbidities, body mass index, physical activity, FIGO stage, histological type, histopathologic grade, menopausal status, oral contraceptive status, parity, residual lesions, total energy intake, dietary change, total fatty acid intake, and cholesterol intake.

** Tests for trend were based on variables containing the median value for each tertile.

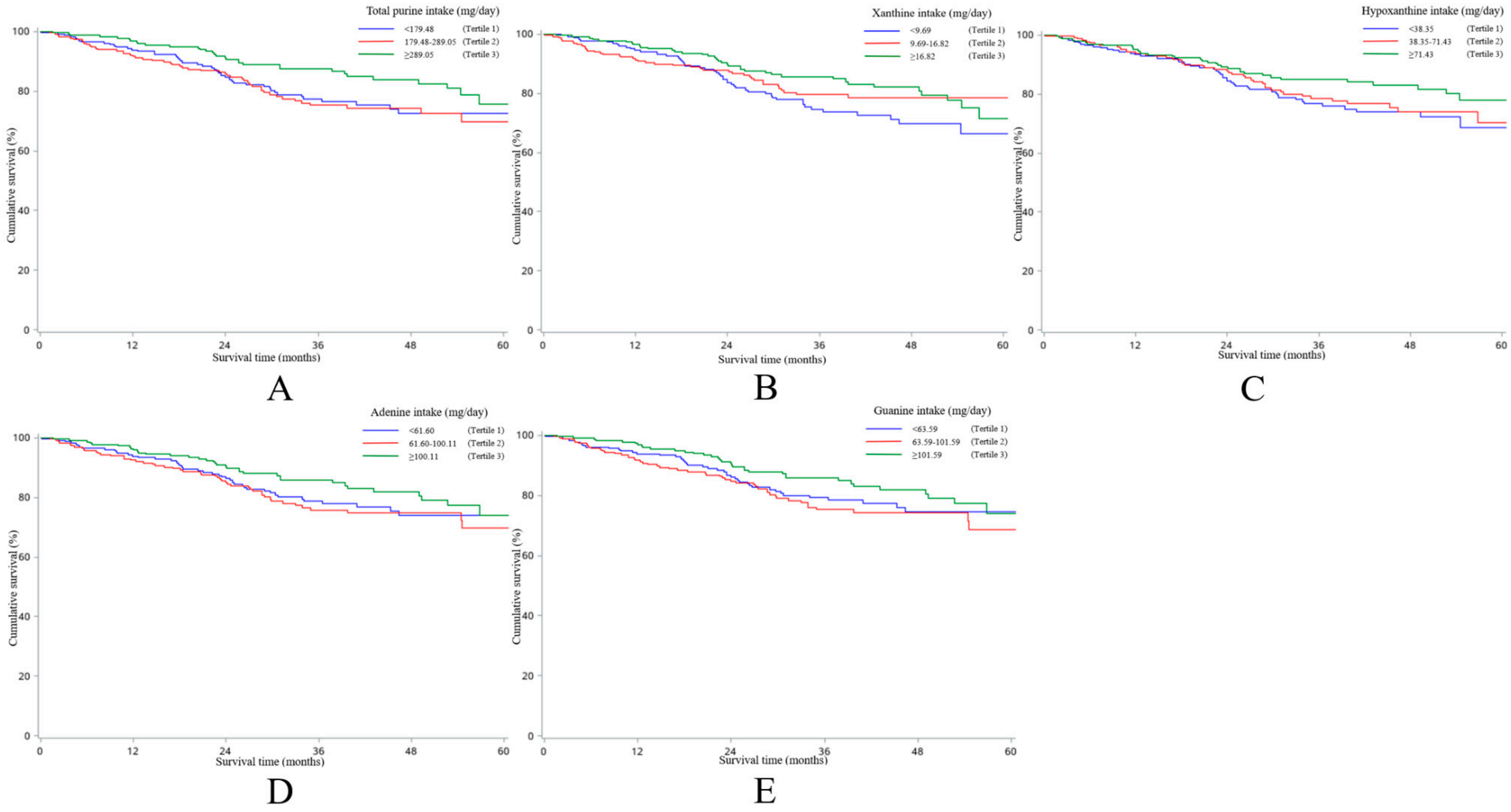

Supplementary Figure S1. Kaplan-Meier survival curves for A: total purine intake; B: dietary xanthine intake; C: dietary hypoxanthine intake; D: dietary adenine intake; E: dietary guanine intake.

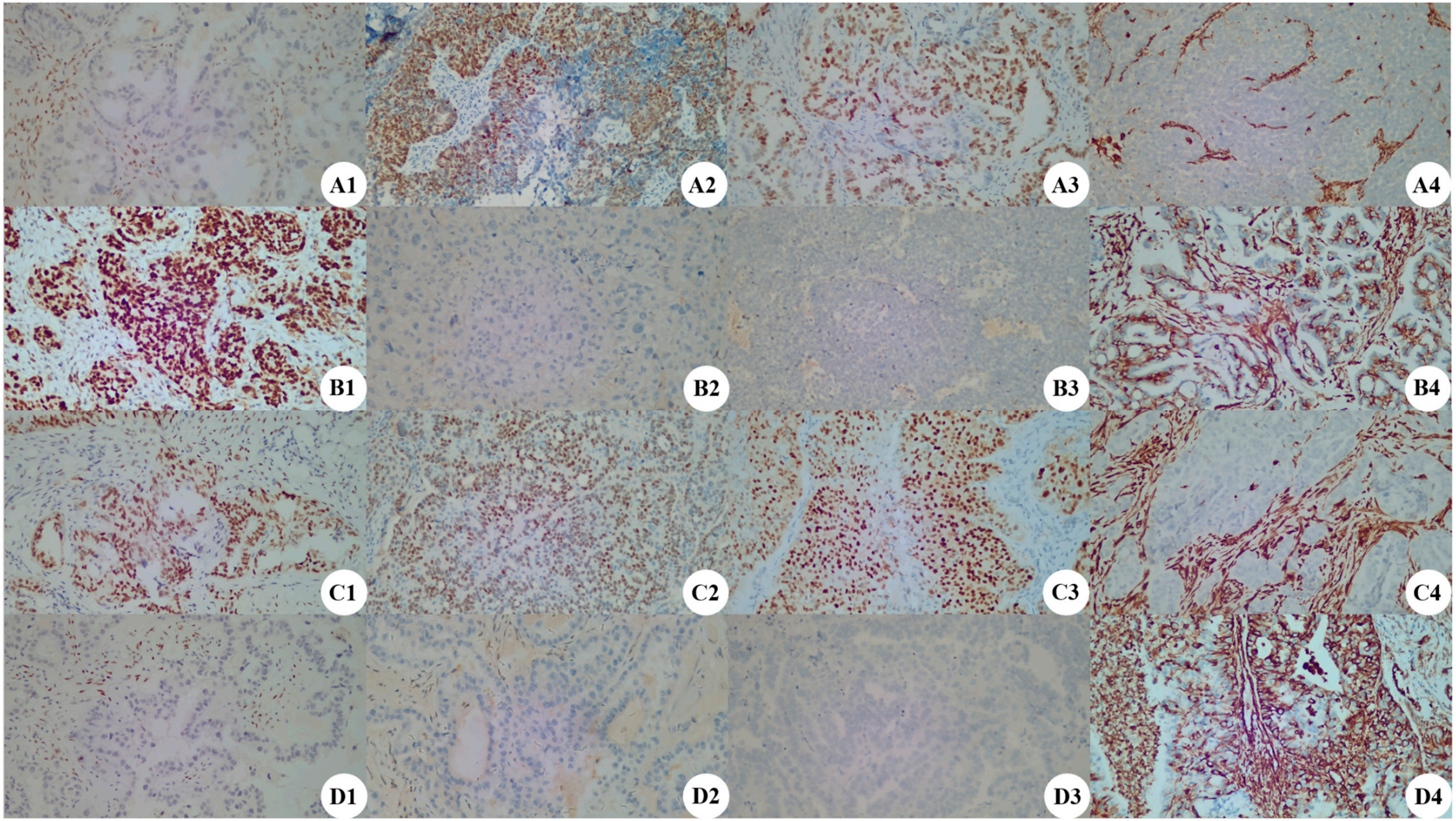

Supplementary Figure S2. Immunohistochemical images of different xanthine intake and hypoxanthine intake levels.

A1: Image of WT-1 protein in patients with high xanthine intake; **A2:** Image of ER in patients with high xanthine intake; **A3:** Image of PR in patients with high xanthine intake; **A4:** Image of Vimentin in patients with high xanthine intake; **B1:** Image of WT-1 protein in patients with low xanthine intake; **B2:** Image of ER in patients with low xanthine intake; **B3:** Image of PR in patients with low xanthine intake; **B4:** Image of Vimentin in patients with low xanthine intake; **C1:** Image of WT-1 protein in patients with high hypoxanthine intake; **C2:** Image of ER in patients with high hypoxanthine intake; **C3:** Image of PR in patients with high hypoxanthine intake; **C4:** Image of Vimentin in patients with high hypoxanthine intake; **D1:** Image of WT-1 protein in patients with low hypoxanthine intake; **D2:** Image of ER in patients with low hypoxanthine intake; **D3:** Image of PR in patients with low hypoxanthine intake; **D4:** Image of Vimentin in patients with low hypoxanthine intake.

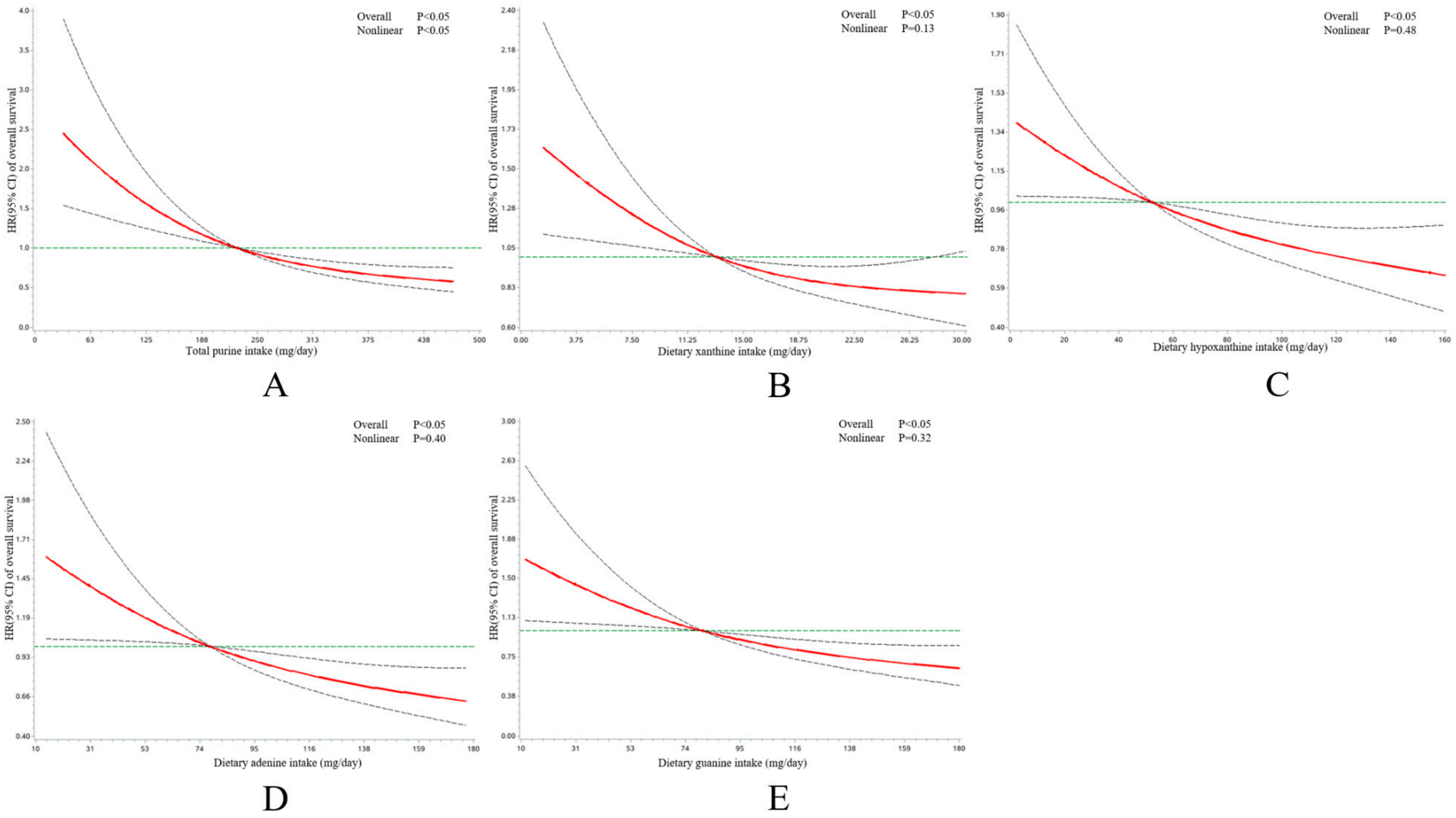

Supplementary Figure S3. HR and 95%CI of mortality among ovarian cancer patients by A: total purine intake; B: dietary xanthine

intake; C: dietary hypoxanthine intake; D: dietary adenine intake; E: dietary guanine intake.

The association was adjusted for HR and 95% CI was calculated through the Cox proportional hazards regression model with adjustment for age at diagnosis, education level, income level, smoke status, alcohol intake, comorbidities, body mass index, physical activity, FIGO stage, histological type, histopathologic grade, menopausal status, oral contraceptive status, parity, residual lesions, total energy intake, dietary change, total fatty acid intake, and cholesterol intake. The red line and dashed line represent the estimated HRs and their 95% CIs, respectively. CI, confidence interval; HR, hazard ratio.
